# Analysis of polyethylene wear in plain radiographs: The number of radiographs influences the results

**DOI:** 10.3109/17453670903487024

**Published:** 2009-12-04

**Authors:** Maiken Stilling, Kjeld Søballe, Niels Trolle Andersen, Kristian Larsen, Ole Rahbek

**Affiliations:** ^1^Department of Orthopaedics, Aarhus University Hospital; ^2^Department of Biostatistics, School of Public Health, Aarhus University; ^3^Orthopedic Research Unit, Department of Orthopedics, Hospital Unit West, Holstebro, Denmark

## Abstract

**Background and purpose** Two-dimensional computerized radiographic techniques are frequently used to measure in vivo polyethylene (PE) wear after total hip arthroplasty (THA), and several variables in the clinical set-up may influence the amount of wear that is measured. We compared the repeatability and concurrent validity of linear PE wear on plain radiographs using the same software but a different number of radiographs.

**Methods** We used either 1, 2, or 6 anteroposterior (AP) hip radiographs of 11 patients from a clinical THA series with 12 years of follow-up, and measured the PE wear with the software PolyWare 3D Pro. Repeatability within and concurrent validity between the different numbers of radiograph strategies were assessed using limits of agreement (LOAs) and bias.

**Results** Observed median wear (range) in mm was 3.4 (1.6–4.6), 2.3 (0.7–4.9), and 4.0 (2.6–6.2) for the 1-, 2-, and 6-radiograph strategies. For repeatability, no bias (p > 0.41) was observed. LOAs around the bias were ± 0.6, ± 0.4, and ± 1.2 mm for the 1-, 2-, and 6-radiograph strategies. For concurrent validity, a bias (± LOA) between all pairwise comparisons was observed (p < 0.02) with 0.8 mm (± 2.5) between the 1- and 2-radiograph strategies, 1.0 mm (± 2.2) between the 1- and 6-radiograph strategies, and 1.8 mm (± 1.2) between the 2- and 6-radiograph strategies.

**Interpretation** The number of radiographs used for wear measurement with a shadow-casting analysis method on plain AP radiographs influences the amount of linear wear measured. Results of PE wear obtained with PolyWare in studies using a different number of radiographs are not comparable.

## Introduction

Polyethylene (PE) wear of more than 0.1–0.2 mm/year is associated with later osteolysis and failure of total hip arthroplasty (THA) (Sochart 1999, Dowd et al. 2000). In vitro simulator wear studies may not reflect the total sum of PE wear seen in vivo, and therefore continuous investigations of PE wear in the clinical setting with matching reports of the clinical outcome are important. Several different methods are currently used to estimate clinical wear after THA, but few comparisons have been made (Collier et al. 2003, Ebramzadeh et al. 2003, Hui et al. 2003, Kang et al. 2003, von Schewelov et al. 2004, Bragdon et al. 2006a, Geerdink et al. 2008). Wear measurements of hip arthroplasty are most accurately performed with radiostereometric analysis (RSA) (Bragdon et al. 2002, von Schewelov et al. 2004, Borlin et al. 2005). RSA, however, which is limited to prospective studies with recordings of stereometric radiographs at all follow-ups, requires an expensive set-up and is not easily established. Consequently, plain radiographs are still used in most descriptions of clinical wear.

Previous studies have shown great variation in wear measurements for specific components, which may in part be caused by intraobserver variance, component and patient factors (Orishimo et al. 2003), pelvic orientation (Collier et al. 2003, Foss et al. 2008), and the radiographic quality (Sychterz et al. 2001). Furthermore, it is unlikely that different methods used to measure PE wear will agree exactly by giving identical results for all individuals (Bland and Altman 1986)—as has also been demonstrated in comparative studies (Collier et al. 2003, Hui et al. 2003, Bragdon et al. 2006a). PE wear results obtained by manual methods are known to have large interobserver variance, and results obtained by manual methods may be difficult to compare directly with results obtained by computerized methods, which have a more predictable accuracy and far better precision (Hui et al. 2003, McCalden et al. 2005).

There is currently no consensus concerning how wear analysis is best performed and presented. Bedding-in of the PE component has led some researchers to favor exclusion of the initial period (months to years) of follow-up (Sychterz et al. 1999a, Hui et al. 2003), but probably the period and magnitude of creep vary between components. Inclusion of the initial period of wear certainly increases the mean measured wear and also the wear rates calculated. This is particularly problematic when comparing wear rates in studies of short-term follow-up versus long-term follow-up. Some research groups recommend analysis of serial radiographs (Sychterz et al. 1997), while others analyze only the latest follow-up radiographs and assume zero wear at baseline (Norton et al. 2002). Few evaluate the precision of their own investigations with the method chosen but rather refer to a specialized laboratory for determination of the precision.

Observations and questions raised in our research group on assessment of clinical wear after hip arthroplasty with a computerized shadow-casting technique (Devane et al. 1995b) inspired us to investigate in greater detail whether analysis of a single, two, or multiple radiographs in the same clinical series of patients would result in different estimates of wear, and if so, how different they would be.

## Materials and methods

### Study design

We measured two-dimensional femoral head penetration into the PE liner in a selected group of 11 patients from a clinical series of 27 patients (28 hips) formerly evaluated for early migration of the femoral stem (Soballe et al. 1993) and later for cup revision, PE wear, and osteolysis (Stilling et al. 2009). The acetabular component used was a hemispherical rim flair screw-fixed Universal Hexloc metal backing (Biomet Inc., Warsaw, IN) with a 10-degree face GUR 415 bar extruded conventional ultra-high molecular weight PE acetabular liner sterilized by gamma radiation in air. The femoral component was a cementless, proximally coated Bi-Metric stem (Biomet). Cobalt-chromium 28-mm femoral heads were used. The acetabular shells ranged in size from 48 to 62 mm, and the PE thickness ranged from 3.39 to 6.47 mm. One surgeon had performed all the operations using a posterolateral approach. All the radiographs had been taken in the same hospital between 1990 and 2003. No specific radiographic protocols other than the standard one for the hospital had been used. The center beam had been aimed at the hip joint (the femoral head).

The 11 patients (6 men, 5 women) from the original group of 28 patients were selected by the criterion of all having 12 years of radiographic follow-up with 6 good-quality AP radiographs and no apparent migration of the cup, as changes in cup angulation have been shown to influence wear measurements with the used software (Collier et al. 2003). The 17 patients not included did not have a full sequence of 6 radiographic follow-ups from baseline to 12 years (for example, due to missing postoperative AP radiographs), or less than 12 years of follow-up because of death or revision. Despite the fact that the postoperative printed radiographs had been stored for almost 15 years, they were in a satisfactory condition and we did not exclude any patients because of insufficient quality of AP images. Cross-table lateral radiographs were also available, but we chose not to include them because of their poor quality and other problems described in the literature (Sychterz et al. 1999b, 2001). The 6 radiographs were taken at the following time points: postoperatively (within days) and 3 months, 6 months, 1 year, 5 years, and 12 years after surgery. 6 hydroxyapatite-coated components and 5 non-hydroxyapatite components were used. Some of the patients had high amounts of wear and some had low amounts of wear.

### Radiographs and software

**Figure 1.**
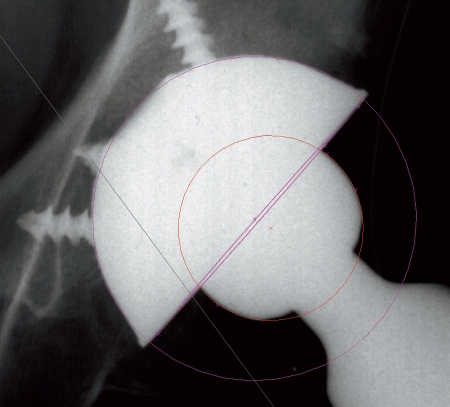
Analysis of PE wear with PolyWare Digital Edition demonstrating digital edge detection.

The AP radiographs were all digitized to tagged image files at a resolution of 300 dots per inch with a transmission-light scanner (Mustek P3600 A3 pro, Irvine, CA). The location of the central ray was estimated by pencilling diagonals between the corners of the rectangular exposure on the radiograph. Analysis was performed with a computerized method featuring a digital edge-detection algorithm to fit circles and ellipses to the peripheral shadows of the femoral head and acetabular component (PolyWare Pro 3D Digital version 5.10; Draftware Developers, Conway, SC) (Figure 1). This technique, developed by Devane et al. (1995a,b), relies on computer-assisted technology to create a 3-dimensional solid model of the acetabular component and femoral head based on back-projection of the radiographs, the femoral head size, and knowledge of the design of the acetabular component (CAD library of various prosthetic brands in the software). Femoral head penetration is then calculated as the difference between vector lengths on subsequent measurements. The stated precision of linear wear with the software version used is approximately 0.089 mm (Devane and Horne 1999).

The quality of the digitized AP radiographs was generally good, and the automatic circle-fitting in the PolyWare wear measurement software only rarely had to be overruled by the manual digitizer tablet. Whenever the edge-detection routines failed to accurately locate peripheral shadows of the acetabular component or femoral head, the observer applied 5 evenly-spaced dots manually on the peripheral shadow of the components. This was the case in 3 of the 198 analyses.

### 3 strategies of wear analysis

For wear analysis, we used 3 strategies commonly reported in the literature and compared the results. Firstly, we analyzed all 6 follow-ups and added the sequential wear between follow-ups to obtain the mean linear wear. Secondly, we analyzed the postoperative follow-up versus the final 12-year follow-up, and thirdly we analyzed only the 12-year follow-up, assuming zero wear at the time of operation. In what follows, the 3 strategies are referred to as PW_6_ (6 radiographs), PW_2_ (2 radiographs), and PW_1_ (1 radiograph).

132 analyses (11 patients × 6 radiographs × double analysis) were performed with the PW_6_ strategy. The mean wear estimates for PW_2_ were based on 44 analyses (11 patients × 2 radiographs × double analysis), and 22 analyses (11 patients × 1 radiograph × double analysis) were performed for the wear estimate of PW_1_.

### Statistics

Repeatability (random variation or precision) of the software package PolyWare was assessed as the standard deviation of the difference (SD_dif-intra_) between two PE wear measurements on the same radiographs for a particular radiograph strategy (PW_1_, PW_2_, PW_6_). According to Altman (1995), we further calculated limits of agreement (LOAs), in this case, LOA_intra_ as (SD_dif-intra_ × ± 1.96). The systematic variation (bias) between the double measurements was estimated as the mean difference between the 2 measurements. The differences between the 2 measurements followed Gaussian distribution (Shapiro-Wilk test (Altman 1995)) and these were tested by a paired t-test. The measures of repeatability (SD_dif-intra_ or equivalent the width of LOA_intra_) of the 3 strategies were compared pairwise by Pitman's test.

Criterion validity defines the correlation of a measurement and an external criterion of the phenomenon under study, while the sub-aspect concurrent validity defines the time-chronological correlation (International Epidemiological Association 1995). Thus, concurrent validity was used for comparison of the 3 strategies of time-chronological wear measurement. For each strategy, we used the average value of PE wear from the double measurements, then estimated the difference between 2 strategies, and finally estimated the standard deviation of the difference (SD_dif-inter_) between these strategies with LOA_inter_ as (SD_dif-inter_ × ± 1.96) (Altman 1995). The bias between 2 strategies was investigated as the difference in mean measured PE wear. It followed a normal distribution (Shapiro-Wilk test) (Altman 1995), and was tested by a paired t-test. The correlation between methods was described by the coefficient of correlation (r).

Statistical significance was assumed at p < 0.05. Intercooled Stata 10.0 (StataCorp, College Station, TX) was used for statistical computations.

## Results

**Figure 2.**
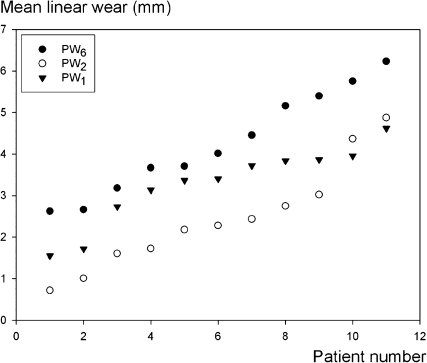
Patients 1 through 11 sorted by increasing magnitude of wear for each of the 3 (1-, 2-, and 6-radiograph) strategies.

Observed median wear (range) for the 11 patients was 3.4 (1.6–4.6) mm, 2.3 (0.7–4.9) mm, and 4.0 (2.6–6.2) mm for PW_1_, PW_2_, and PW_6_ (Figure 2 and Table 1).

**Table 1.** Repeatability of radiographic double wear measurements within the strategies

Analysis strategy	Median (range)	SD_dif-intra_^a^ (mm)	Bias ^b^ ± LOA ^c^	95% CI ^d^ (mm)	p-value ^e^ (mm)
2D measurements of wear					
PW_6_^f^	4.02 (2.63–6.24)	0.61	–0.08 (± 1.22)	–0.49 to 0.33	0.7
PW_2_^g^	2.28 (0.72–4.88)	0.18	0.05 (± 0.37)	–0.08 to 0.17	0.4
PW_1_^h^	3.40 (1.55–4.62)	0.28	–0.02 (± 0.56)	–0.21 to 0.17	0.8

^a^ SD_dif-intra_ is the random variation within a strategy comparing double measurements. ^b^ Bias: systematic variation within a strategy. ^c^ LOA: Limits of agreement around the bias (95% prediction interval = SD_dif-intra_ × 1.96). ^d^ 95% confidence interval for the bias. ^e^ p-value (paired t-test) for bias between strategies. ^f^ PW_6_: PolyWare PE wear analysis using 6 follow-up radiographs. ^g^ PW_2_: PolyWare PE wear analysis using the postoperative radiograph and the final follow-up radiograph. ^h^ PW_1_: PolyWare PE wear analysis using only the final follow-up radiograph.

**Figure 3.**
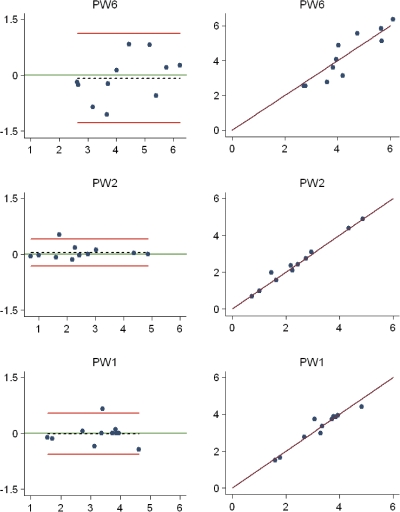
Bland-Altman plots (left) and scatter plots (right) with lines of equality for repeatability measures for each of the three strategies.PW_6_: PolyWare using 6 follow-up radiographs; PW_1_: PolyWare using only the final follow-up radiographs; PW_2_: PolyWare using the postoperative and the final follow-up radiographs. In the Bland-Altman plots (left-hand panels): x-axis, average of 2 measurements; y-axis, difference between 2 measurements (y = measurement 1 – measurement 2); red lines, 95% limits of agreement; dashed line, bias from 0; long solid green line, y = 0 line; dots, individual double measures. In the scatter plots (right-hand panels): x-axis, first measurement; y-axis, second measurement; maroon lines, lines of equality.

For repeatability, no bias was observed (p > 0.41). LOAs around the bias were ± 0.56, ± 0.37, and ± 1.22 mm for PW_1_, PW_2_, and PW_6_, respectively. SD_dif-intra_, bias, LOA around the bias, 95% confidence interval (CI) around the bias, and p-value for paired t-test are given in Table 1 and Figure 3. The relative repeatability was different between PW_1_ and PW_6_ (p < 0.001), and between PW_2_ and PW_6_ (p = 0.02) (Table 2).

**Table 2.** Comparison of repeatability and concurrent validity between strategies

	Repeatability			Concurrent validity		
	Relative repeatability^a^	p-value ^b^	SD_dif-inter_^c^(mm)	Bias ^d^ ± LOA ^e^ (mm)	95% CI of true bias ^f^ (mm)	p-value ^g^
Absolute 2D measurements						
PW_6_^h^ vs. PW_2_^i^	3.34	< 0.01	0.59	1.81 (± 1.19)	1.41–2.21	< 0.001
PW_6_ vs. PW_1_^j^	2.17	0.03	1.12	1.00 (± 2.24)	0.24–1.75	0.01
PW^1^ vs. PW_2_	1.54	0.21	1.26	–0.81 (± 2.52)	0.05–0.49	0.02

^a^ Relative repeatability: ratios of variance. ^b^ p-value: test of variance between strategies (Pitman's test). ^c^ SD_dif-inter_: random variation from the 2 different strategies. ^d^ Bias: systematic variation between strategies. ^e^ LOA: Limits of agreement around the bias (95% prediction interval = SD_dif-inter_ × 1.96). ^f^ 95% confidence interval for the bias. ^g^ p-value (paired t-test) for bias between strategies. ^h^ PW_6_: PolyWare PE wear analysis using 6 follow-up radiographs. ^i^ PW_2_: PolyWare PE wear analysis using the postoperative radiograph and the final follow-up radiograph. ^j^ PW_1_: PolyWare PE wear analysis using only the final follow-up radiograph.

**Figure 4.**
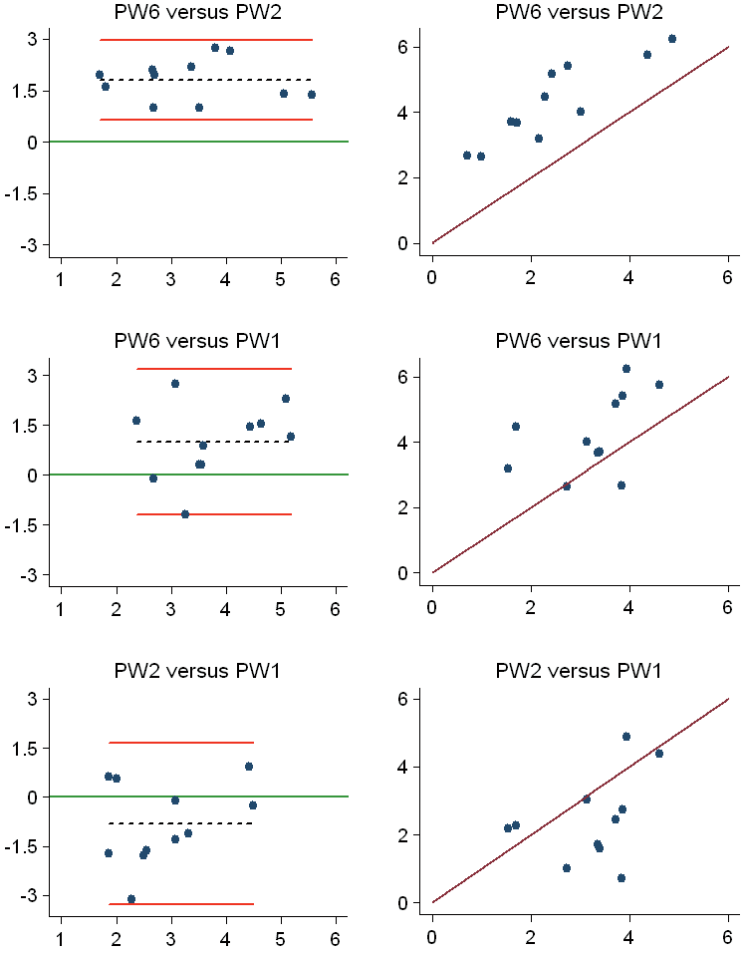
Bland-Altman plots (left) and scatter plots (right) with lines of equality for concurrent validity between the three strategies. PW_6_: PolyWare using 6 follow-up radiographs; PW_1_: PolyWare using only the final follow-up radiographs; PW_2_: PolyWare using the postoperative and the final follow-up radiographs. In the Bland-Altman plots (left-hand panels): x-axis, average of the measurements of 2 strategies; y-axis: difference between measurements of two strategies; red lines, 95% limits of agreement; dashed line, bias from 0; long solid green line, y = 0 line; dots, individual double measures. In the scatter plots (right-hand panels): maroon lines, lines of equality.

For concurrent validity, significant bias between all pairwise comparisons was observed (p < 0.02) with 0.81 (LOA: ± 2.52) mm between PW_1_ and PW_2_, 1.00 (± 2.24) mm between PW_1_ and PW_6_, and 1.81 (± 1.19) mm between PW_2_ and PW_6_ (Table 2 and Figure 4). The correlations were 0.39, 0.50, and 0.89 for PW_2_ and PW_1_, PW_6_ and PW_1_, and PW_6_ and PW_2_, respectively.

## Discussion

The purpose of this study was to determine whether the number of radiographs used for analysis with a digital shadow-casting wear analysis technique would influence the wear results. No distinction between creep, articulate wear, and backside wear could be made with the method of wear analysis we used, and since all cups were unrevised, the true amount of wear remains uncertain.

The magnitude of wear obtained with all 3 strategies of wear analysis was high and well above the 0.1–0.2 mm/year threshold of linear PE wear described to cause later complications of osteolysis and revision (Sochart 1999, Dowd et al. 2000). We have evaluated PE wear to the final follow-up (death, revision, or 12-year) in all patients of this formerly randomized patient group in a different study, in which we further addressed the resultant complications of excessive osteolysis and revisions and discussed reasons for the magnitude of PE wear (Stilling et al. 2009). The study group consisted of both Ti-coated cups and HA-coated cups, and we have shown a statistically insignificant but clinically relevant difference in total PE wear between the Ti and HA groups of 3.8 mm and 4.8 mm after an average of 11 years (Stilling et al. 2009). The present study investigated the degree of PE wear in a random group of patients and in a range that was relevant for the software used (Hui et al. 2003), and we do not believe that the difference in magnitude of wear with Ti and HA components would affect the conclusions from these measurements.

We observed large differences in measured median PE wear in the same patients between the 3 strategies, and the PE wear estimated with the 6-radiograph strategy was almost twice that observed with the 2-radiograph strategy. This bias was consistent for the individual measurements (Figure 2), except for 2 values close to wear-through of the liner. Repeatability was found to be best for PW_1_ and PW_2_, with LOA around the bias of 0.6 and 0.4 mm, which was better than for PW_6_. One explanation for the rather high random variation in repeatability observed for PW_6_ could be the inherent problem that each of the 5 PE wear estimations in this strategy contributes with positive values and variances are summed up from examination to examination. It therefore seems that a multiple-radiograph strategy is best when monitoring the development of wear over time, and less favorable when is comes to a precise estimate of wear at a given time point. Regarding concurrent validity, we observed a large systematic variation of 1.8 mm with a clinically acceptable random variation (± 1.2 mm) between PW_2_ and PW_6_. However, the random variation between PW_1_ and the other radiograph strategies exceeded ± 2 mm. The systematic variation can be corrected for if known, whereas this is not possible for the random variation, and thus it seems that clinical measurements obtained with PW_2_ were similar to those obtained with PW_6_, with a correlation of 0.89 mm. We were rather surprised to find a low concurrent validity between PW_1_ and PW_2_, as, in theory, the random variation for both should have been small. The final follow-up radiograph was the same in both strategies; thus, the difference must have arisen from the handling of the starting point by the software. For PW_1_, the software decides the position of zero PE wear (baseline) from CAD-derived knowledge of the cup component and size, along with information about the femoral head size, whereas with PW_2_ the actual baseline position of the cup and head, as estimated from the baseline radiograph, is used for the calculation of PE wear. More research is needed to determine what contributes to the differences between PW_1_ and PW_2_, and to explain whether this is only problematic for the Universal component implant brand. In addition, it is not known which strategy (PW_1_ or PW_2_) better reflects the true wear.

The accuracy and precision of clinical PE wear estimates depend on several variables, including patient factors (Schmalzried and Huk 2004), radiographic quality (Sychterz et al. 2001), assumptions of linear wear patterns (Yamaguchi et al. 1999), hip angulations (Collier et al. 2003, Foss et al. 2008), the wear analysis method used (Bragdon et al. 2006a), intraobserver variance (Engh, Jr. et al. 2002), and manufacturing tolerances of acetabular components (Hui et al. 2003). Plain AP radiographs used for wear analysis are not calibrated (position coordinates), and in retrospective studies radiographs are often not obtained according to a standardized protocol. The clinical positioning of patients with the risk of slight changes in hip angulations between radiographic follow-ups has been shown experimentally to influence wear results (Collier et al. 2003, Foss et al. 2008). The greater the change in angulations between follow-ups, the larger the magnitude of wear measured (Collier et al. 2003). A plausible theoretical explanation for this is that the radiographic shadows of the components vary with angular displacements, making the basis for automatic edge detection different between follow-ups. Recently, a mathematical correction algorithm has been suggested to make 2-dimensional wear measurements in plain radiographs less sensitive to radiographic projection differences and to approximate 3-dimensional “true” linear wear values obtained by RSA (The et al. 2008). Radiographic projection differences are difficult to control and offer some explanation for the differences in magnitude of measured PE wear by use of few rather than multiple radiographs, which we observed. This observation further stresses the use of a strict protocol for patient positioning for standard hip radiographs. The clinical radiographs in our clinical study were all obtained according to the standards of one radiology department, although not according to a specified study protocol. Thus, the leg was not placed in a soft foam positioner or rotation-stabilized by a fixture, and this most likely affected the projection between radiographs obtained over a long follow-up period.

Despite such problematic issues of estimating PE wear in clinical studies with plain radiographs, PolyWare has been validated for both research and clinical use, and wear measurements have been described to correlate well with measurements of true wear (Ebramzadeh et al. 2003, Hui et al. 2003). Furthermore, digital wear-analysis methods using plain radiographs are far more precise than the early manual methods (Livermore et al. 1990), although radiostereometric analysis is the most precise tool (Borlin et al. 2002, Bragdon et al. 2002, von Schewelov et al. 2004). Repeatability (precision) of linear 3-dimensional femoral head penetration with PolyWare assessed with phantom images is reported to be between 0.10 mm (Collier et al. 2003) and 0.15 mm (Kang et al. 2003). We determined the intraobserver precision with double analysis (using the same images) and found that analysis of 6 radiographs resulted in a higher mean difference (0.08 mm) than with analysis of 2 radiographs (0.05 mm), and 1 radiograph (0.02 mm). This is not surprising because analysis of more radiographs would be expected to introduce more variance due to radiographic projection and quality.

Wear analysis with PolyWare is based on a single wear vector and is likely to underestimate the true wear in vivo (Hui et al. 2003), which has been shown to occur multidirectionally (Yamaguchi et al. 1999). However, analysis of AP radiographs has been shown to provide a sufficient estimate of the major wear vector (Sychterz et al. 1999b) in THA, and although a 2-dimensional technique on plain radiographs slightly underestimates wear (Hui et al. 2003), repeatability is better than that obtained with 3-dimensional techniques, which often rely on lateral radiographs of suboptimal quality (Sychterz et al. 2001). Other causes of wear underestimation may be that the PE wear tract is not a tight cylinder around the femoral head (Devane et al. 1995a). Furthermore, there are no guarantees that the head will be located at the deepest point of the wear tract at the time of radiography (Devane et al. 1995a). Several PE wear studies have addressed the potential of weight-bearing supine radiographs for PE wear analysis and the conclusion has been that the measured differences in PE wear between weight-bearing and non-weight-bearing radiographs are of no clinical relevance (Martell et al. 2000, Bragdon et al. 2006b, von Schewelov et al. 2006).

Much attention has been given to definition, calculation, and exclusion of the initial and delimited period in clinical follow-up based on theories of creep or bedding-in of the PE liner (Sychterz et al. 1999a, Glyn-Jones et al. 2008), but no consensus has been reached. Creep may depend on various factors, including acetabular component design, activity of the patient (friction heating), and the type or quality of PE. “True in vivo wear” can be described in retrieval studies by coordinate-measuring machines (CMM), and while this offers an accurate estimate of the articulate wear, including creep, backside wear cannot be quantified (Hui et al. 2003). It is thus problematic to correlate the defined “true in vivo wear” obtained by CMM with radiographic measurements of wear that include both articulate and backside wear, and it becomes even more complicated when the first postoperative period is excluded because of theories of creep (Hui et al. 2003). In addition, the exclusion of a variable period of “bedding-in” (6 weeks to 2 years) in some but not all studies inevitably results in different magnitudes of reported wear and wear rates, even though efforts are made to calculate intercepts and the steady-state wear. Thus inter-study comparisons of PE wear are difficult, and there is a need for a standardization guide for the presentation of PE wear results and precisions.

The radiographs used in our study were all printed films digitized for computed wear analysis. Physical degradation and varying resolution may have influenced our wear analyses, because the first radiographs were obtained in 1990. The PW_2_ and PW_6_ radiograph strategies involved both old and new radiographs, whereas the PW_1_ strategy was based on only one recent radiograph of potentially superior quality. As we did pay attention to the radiographic quality, only the AP radiographs were selected and they were all judged to be of good quality in terms of visual implant borders. In support of this, the automated digitizer system for the software (PolyWare) only failed and had to be manually overruled a total of 3 times in 198 wear analyses. Future improvements with direct digital radiographs may improve the precision of PolyWare, but currently the software is recommended only for series of substantial wear, such as UHMWPE liners in long-term follow-up or populations of failed implants (Hui et al. 2003); due to the random variation observed, large sample sizes are to be recommended.

RSA is the most accurate tool for wear analysis and it could be regarded as the gold standard for clinical wear analysis (Bragdon et al. 2002, von Schewelov et al. 2004). Unfortunately, we did not have the stereo radiographs needed to compare the wear results of the 3 strategies using plain radiographs with RSA. Studies on patient series with adequate long-term plain and stereo radiographic follow-ups should focus on this matter. On the basis of our findings, analysis of the same number of radiographs per patient should be attempted in clinical studies assessing PE wear using plain AP radiographs.

In conclusion, our results show that there are indeed limitations to comparing mean PE wear results based on analysis of different numbers of plain AP radiographs. Inter-study results of PE wear with PolyWare software using 2 or multiple serial radiographs correlate well and seem comparable. However, care should be taken when mixed strategies are used, and we do not advise comparing PE wear in groups by assessing an unequal number of available radiographs per patient.
